# Toward a Cultural Psychology of Conspiracy Theories: A life-narrative analysis of Flat Earthers

**DOI:** 10.1007/s12124-024-09857-5

**Published:** 2024-07-26

**Authors:** Kirstine Pahuus, Maja Sødinge Jørgensen, Brady Wagoner

**Affiliations:** 1 Aalborg University DK, Aalborg, Denmark; 2 University of Copenhagen DK Aalborg University DK, Aalborg, Denmark

**Keywords:** Flat Earth, Conspiracy theories, Worldviews, Narratives, Motives

## Abstract

While the idea of a flat earth may seem absurd in the twenty-first century, there is today a large and growing number of people who believe it. Who are these people and what animates their belief? In answering these questions, this article aims to articulate a cultural psychological approach to conspiracy theories. This is advanced through an in-depth narrative analysis of three individuals' life stories concerning before, during, and after the transition to the new belief. Thus, rather than starting from the typical look at what socio-demographic factors predict conspiracy beliefs, we start from a nuanced look at flat earth believers' own life worlds. We show how different individual motives (epistemological, social and existential) and knowledge systems (scientific, religious, societal) come together in individuals’ adoption and reconstruction of conspiracy theories so that they resonate with believers’ personal lives. Most importantly, flat earth theory offers people a comprehensive vision that places human beings at the center of the universe and provides arguments for how life is meaningful. However, we show that this is reached through different pathways in accordance with people's unique life histories and challenges.

## Introduction

What if the Earth is flat? Perhaps science has miscalculated and the falsehood has been perpetuated by unscrupulous actors, working behind the scenes? Starting from this radical skepticism has the potential to turn many accepted worldviews upside down, as it has for a growing number of people called Flat Earthers. Flat Earth (FE) theory is an alternative explanation of the complex interconnections of the universe that starts from the assumption that the world is a disc, in sharp opposition to today’s scientific consensus of spherical cosmology (see Nothaft, 2017; Kuzii & Rovenchak, 2019; Loxton, 2019). If the world is in fact flat then various government agencies (e.g., National Aeronautics and Space Administration, government agencies, and educational systems) must be false (Andert et al., 2018; Van Prooijen et al. 2020).

The present study aims to examine the thought styles and underlying motives that lead people to believe in FE, within a particular cultural tradition. FE theory is approached as a pattern of thinking characteristic of conspiracy theories (CT), that is, “nothing happens by accident; nothing is as it seems; and everything is connected” (Barkun, 2013, pp. 3–4). Taking a cultural psychological perspective, we argue that research on CT in general and FEs in particular needs to go beyond the typical correlational study of belief with a host of other demographic and behavioral measures (for a recent review see Pilch et al., 2023) to explore how beliefs are related to a person as a complex psychological system within a complex cultural context. The analysis here will thus highlight both the content of beliefs and show how personal, social, and cultural factors come together in the concrete lives of three FEers. This will involve analyzing and comparing the contextualized pathways by which they incorporate conspiracy beliefs into their lives, following their progressive 'internalization' and how this relates to life events, other beliefs, cultural symbols, and different psychological motives. In other words, we will follow the framework of cultural psychology to explore the mutual constitution of persons and their social-cultural worlds (Shweder, 1991; Valsiner, 2007).

## Flat Earth Belief as a Conspiracy Theory

Conspiracy theories (CT) are responses to the complexity of the world and are abundant in times of ambiguity and rapid changes (van Prooijen, 2011). They are attempts to explain seemingly random events through a secret plot enacted by powerful and evil agents. This points to a distinctive ‘mentality’ or ‘thought-style’ for making sense of the social world, which is socially embedded within intergroup relations and their history (Moscovici, 1987). Goertzel’s (1994) early findings suggested that the best predictor of belief in any CT was belief in other CTs. He defined it as a monological way of thinking that expressed a “closed” mindset, as arguments outside CTs were not taken into account. Recent findings argue that CT should rather be understood dialogically, since CT believers engage in a dialogue with their context (Franks et al., 2017; Hall 2020). For example, they advance arguments that are based on semantic and pragmatic relations to non-conspiratorial beliefs (scientific or otherwise) that are marshaled to support the CT.

Similar to other conspiracy theories, modern FE Theory does not limit itself to factual explanations, but also incorporates a skepticism about the credibility of modern media (Aupers, 2020), the structure of society (Brotherton and French 2015), and general complexity of explanations (Krekó, 2020). It differs from other CTs, however, in its reliance on common sense arguments, based on visual experience and the results of homemade models (Břízová et al. 2018). By contrast, understanding the Earth’s spherical form (aside from images of the Earth from space) requires complex mathematical models and thus trust in experts’ knowledge. The homemade models that contradict expert knowledge led to skepticism about whether people in power are hiding the truth from the majority. If the elite would deceive people about the Earth’s shape, what else would they hide? It is thus not surprising that belief in CTs coincides with disbelief in many mainstream narratives (Wood & Douglas, 2015).

Social media has played a key role in the dissemination of misinformative and CTs (Paolillo, 2018) and has been particularly crucial to the propagation of FE theory. Visual arguments for FE on YouTube (Olshansky et al. 2020) and written arguments on Twitter (Melo et al. 2020) have been shown to have a strong impact on belief, although Landrum et al. (2021) suggest that low scientific knowledge and high conspiratorial mentality are key factors in susceptibility to FE arguments online. Similarly, Georgiou et al. (2022) found that higher levels of scientific reasoning and belief flexibility were negatively associated with CT beliefs. Given the complexity of knowledge in modern society, it is important to approach beliefs sensitively; CTs are easy to put forward, but difficult to disprove (Goertzel, 2010). While much research has addressed FE theory by looking at how social media behaviors influence conspiracy theorists, it is also essential to examine the psychological processes that prepare the way for its adoption in the first place.

From a psychological approach, Douglas et al. (2017) have identified three interrelated *motives* associated with conspiracy belief: *epistemic* (understanding one's environment), *existential* (having control over one's environment), and *social* (maintaining a positive image of oneself and the social group one is part of). The epistemic motive includes various cognitive biases identified as making people susceptible to CTs, such as illusory pattern perception (Van Prooijen et al., 2018) through higher-order processes in which people jointly construct an image of the world, as studied by social representations theory (Moscovici, 1981). The existential motive highlights the fact that belief in CTs is highest in situations of unpredictability and uncertainty, and where people feel powerless and lack trust in authorities (Douglas et al., 2019). Existential threat triggers sensory processes and a search for explanations, a void CTs step into. CT belief is consistently much higher among marginalized groups, living under conditions of threat, distrust, and powerlessness (Hornsey, 2020). Finally, the social motive points to how marginalized groups and individuals can become important actors as champions of the truth in a world where everyone has been lied to. Imhoff & Lamberty (2017) suggest that CT belief can be understood as a means of achieving a sense of uniqueness.

## Society and Religion in Conspiracy Theories

A cultural psychological approach requires us to consider—in combination with individual motives—CTs as part of a broader system of culture, society, and history. This societal aspect can fruitfully be approached through social representations theory, which explores how social reality is jointly constructed between people on the basis of previous understandings within a cultural tradition (Moscovici, 1981). First, like other cultural psychological theories, it addresses the complex, dialectical relationship between social knowledge and individual cognition. This highlights the fact that people make sense of and use the same CT in different ways, which will partly be a function of their group affiliations and partly personal factors. Second, all knowledge and belief are situated within a tradition. CTs are not created *ex nihilo* but build on existing ideas and often previous conspiracy theories. Moscovici (2020) suggested one of CT’s principal features is its *Urphänomen*, meaning that through history different CTs tend to emerge from a common spring, as can be seen in the centuries of CTs relating to Jews, Freemasons, and Illuminati. Third, it highlights ‘cognitive polyphasia,’ the idea that different knowledge systems and their thought styles co-exist in the same society and even the same individual. Knowledge is socially organized into different social contexts, which in turn interact and mutually enrich one another. Other cultural psychological approaches concur in highlighting the heterogenous and distributed nature of culture, in contrast to cross-cultural psychology’s comparison of homogenous national averages of some task. In the present context, this helps us to explore how CTs are related to other belief systems and their corresponding thought styles.

While relationships to scientific thinking have already been noted in the context of FTers use of homemade models and experiments, it is also important to mention parallels between CTs and religious beliefs. Although there is no definitive definition of religion, we follow William James (1902) in approaching it as belief in an ‘unseen order’ which gives meaning and value to life when we harmonize ourselves to it. However, CTs are only *quasi-religious* because the people who believe in them do not possess the institutional features of organized religions (Franks et al., 2013), which is an essential feature of sociological definitions of religion like Durkheim’s (1912/1995). Many elements of religion are selectively reconstructed and integrated into CTs but are done so in a fluid and context-dependent way, including specificities of both the religion and CT in question. For example, FE theory contains many religious elements that can be traced back to the Old Testament (Allegro, 2017), including passages in which the earth is described as flat.

Similarly, the dualistic notion of good and evil that is key to the Judeo-Christian tradition (and can be traced back to Zoroastrianism), also figures centrally in FE Theory. In both, the good forces will often fight the evil forces to restore order (Dyrendal et al., 2018; Oliver & Wood, 2014). Religious narratives typically contain characteristic representations to emphasize this notion, such as ‘God ‘, ‘Satan ‘, ‘heaven ‘¸ and ‘hell’. CTs generally typically also use some combination of these in an attempt to explain events as the result of the machinations of powerful, secretive organizations of conspirators (Keeley, 2018). The dualistic nature of CTs is based on the notion that evil forces are holding back information from the public, with an intent to deceive and control the population. The reasoning behind this notion is that the Elite must have a reason to hide the information: If the information is not harmful there would be no sense in concealing it. Religious representations also serve a social purpose, as they categorize people in society and draw a clear distinction between who is good and who is evil.

In addition to the contents, CTs and religion serve similar motives. First, they are both rooted in the need for a framework that can overcome challenges that scientific explanations, for example, are unable to address (Robertson et al., 2018). This includes providing the comfort of knowing that tragic events occur for a reason (Keeley, 1999)—that is, the notion of *theodicy* (Dyrendal et al., 2018). Furthermore, CT and religious belief both strengthen in times of uncertainty, as a form of compensatory control which substitutes a perceived lack of personal control for an external source of control (Douglas et al., 2017). Whereas religions describe this external source as God, Satan or Karma, CTs typically focus on an evil Elite or minority. Believers then cast themselves into a role that aligns with the good forces against the evil ones. Another strategy of creating a theodicy is by placing God as the almighty power, who is ultimately bigger than the Elite, meaning that the evil acts performed by them are part of God's plan. In short, religious belief and CT belief function as a means to affirm the world as a place that can be known and controlled (Wood & Douglas, 2018).

In the present study, we aim to explore how different motives and cultural systems operate together in unique individual cases and through time. To do so we look at how individuals personalize aspects of collective culture (Valsiner, 2014), understood as a system of social representations (Duveen, 2007). Culture operates here on personal and social levels simultaneously (Obeyesekere, 1980): The former speaks to a person's deep life history and motivation, while the latter concerns how it is understood and positions one vis-a-vis others in society. More specifically, we offer an empirically based contribution to how different systems of belief coalesce in FEers, with the purpose of expanding the present understanding of the people behind FE as well as to nuance the theoretical understanding of CT thinking. In what follows, we will present three different life story narratives to this end.

## Methods

### Recruitment

Conspiracy theorists are difficult to observe, due to their fundamental distrust towards science, institutions, and society, as well as a general fear of being misunderstood (Hall, 2020). Moreover, they often view universities as part of the problem, which can complicate the recruitment of willing participants (Wood & Douglas, 2015). With this in mind, we formulated a request for participants on Facebook stating that we were looking for members of the Flat Earth Society (FES) willing to share their experience with the FE theory and general life story, emphasizing that our intentions were to understand their way of thinking and not to be critical of the theory itself. We aimed to post this description in Facebook groups associated with FE, but quickly encountered an access problem as most of the groups were private. When we requested access and permission to share the post, we generally received rejections.

The following week a few administrators granted us access to the forums, where we were able to communicate with the group members. Within a few days of our post going live, we experienced a great deal of interaction, but no one volunteered to be interviewed. Some helpful members shared their insights on the theory and community. One member pointed out that some believers of FE do not wish to be associated with the FES and suggested changing the post to make it broader. We followed this tip and to our delight, the following day two people contacted us on Facebook about participating in the research, on the premise that they would not be associated with FES which we assured would be the case. The following week another person contacted us by email expressing his interest, and with that, we succeeded in recruiting three participants.

Going into the field of interest we were cautious of using the terms “conspiracy theory”, “conspiracy theorist” and “Flat Earther”, as these can imply judgment and critique and are often used by the majority to delegitimize the theories and the people who believe in them (Douglas et al., 2019; van Twist and Newcombe 2018; Dyrendal et al. 2018). In meeting our participants, we were mindful of the connotations attached to the terms and we asked each of the participants if they had any alternative term they preferred us to use during the interview. None of the three participants had any objections and during our conversations it became clear that they all referred to themselves as Flat Earthers and used the word “conspiracy theory” when talking about their beliefs. These curious observations will be elaborated on in the discussion.

### Interviews

The semi-structured interviews were set up to encourage participants to narrate their individual life-stories, while also prompting reflection on their own perceptions and experiences (Bailey-Rodriguez et. al., 2019). The interview guide consisted of open questions about their lives before and after encountering the FE theory, which allowed them to select which parts of their lives they found worth mentioning. We phrased the questions in such a way to invite the answers to appear as a coherent narrative with a beginning, middle, and end (Flick, 2018). This method enables a reciprocal conversation between the participant and interviewer, including follow-up questions for greater accuracy and the opportunity to expand on particular points presented by the participants (Kallio et al., 2016; Whiting, 2008). This is of course a retrospective account of their life experiences, which is subject to constructive remembering (Bartlett, 1932; Wagoner, 2017). As such we treat it as expressing what is psychologically active and relevant for them now, rather than providing a faithful account of events as they happened.

All interviewees gave consent to be recorded and for the data to be used for this study in an anonymized form. The research was conducted in accordance with APA ethical principles. A special IRB approval not required in the Danish system, where the study was conducted. The interviews were carried out in English and Danish respectively according to the participants' origin and ranged between 45–90 min. All three interviews took place in March 2021 using Zoom and were transcribed to capture the context and flow of the conversation, using minimal punctuation (Bailey-Rodriguez et al. 2019).

### Analysis

We adopted a case study approach in order to capture the complexity of individual experiences in context (Paparini et al., 2021). This approach contains a high level of detail and in-depth descriptions at a microlevel and through time (Crowe et al., 2011). The method is often criticized as not providing a basis for generalization because of the small sample size (Flyvbjerg, 2006; Yin, 1984). However, this is to misunderstand the nature of generalization. From a nuanced understanding of the phenomenon (Paparini et al., 2021; Zainal, 2007), case studies aim to generalize to theoretical models that can be tested against other single cases (Valsiner, 2017). In relation to CT, we aim to show how the different motives identified by Douglas et al. (2019) become important and are stressed at different times and to different ends. Thus, the analysis goes beyond Douglas's abstract typology to show how they play out in concrete individual lives and through time.

Further tools of analysis were adopted from a narrative approach, which focuses on life-changing situations, worldviews and self-perceptions (Flick, 2018). Narratives play a key role in people’s construction of meaning and identities (Bailey-Rodriguez, et. al., 2019). McAdams (2001) suggests that identity is created through a coherent self-narrative, in which plots and characters are of particular importance. The life story connects the individual's past, present, and future, and thus helps to create a sense of a continuous, meaningful life. In the case analyses, we highlight people’s sense of life’s ups and downs through McAdams (2001) categories of ‘high points’, ‘low points’, and ‘turning points’. Furthermore, we stress ‘symbols’ and ‘themes’ that come to the fore of participant’s life stories and give structure to them (Gregg, 1998). These are particularly important in understanding the specific role played by scientific arguments and religious elements in CT belief systems, their compartmentalization and interrelations in each case. We started with a very broad approach in the analysis of the interviews: After several readings, we focused on elements that were significant in relation to the participants’ worldviews. We wanted to understand how the social context and the knowledge systems coalesced to form their personal belief systems.

In the following section these three narratives will be presented as individual cases, each contributing to a broader understanding of the phenomenon of identifying as a FEer. Our first participant has a strictly scientific and conspiratorial approach to FE, our second informant draws on both scientific, religious elements and conspiratorial questions, and our third participant presents a primary use of religious elements as well as conspiratorial questions to make sense of the theory. All three participants originate and live in countries which are predominantly Christian, which is reflected in the way some of them interpret FE in relation to religion.

## Three Case Studies: Variations on the Conspiracy Worldview

### Participant 1

P1 is a Danish man in his 50 s, whose wife divorced him due to economic pressures during the financial crisis in 2008. Post-divorce he moved to Norway where he continued to experience financial difficulties and as a result, was unable to visit his family in Denmark during Easter (*existential motive*). The recurring economic crisis represents a *low point* in his life, which came to dramatically impact his worldview. P1 describes that the majority of his time was spent browsing the internet, because he was not able to socialize with his children. On the internet, he encountered the FE theory for the first time. P1 quickly became engrossed in the theory and describes how he eagerly pointed a telescope at the horizon with the aim of measuring the curvature of the Earth, pointing to a scientific approach to the theory. Afterward, he undertook numerous experiments and calculations that yielded results different from those reported by science. Based on his own research (highlighting the *epistemic motive*), P1 concluded that the Earth must be flat, which represented a major *turning point*. P1 describes how what he initially considered to be a strange thesis suddenly constituted his cosmological worldview.

Soon P1 became an administrator in a Danish FE Facebook group. The group and P1 received much media coverage, an experience that constituted a *high point* in P1's life. Subsequently, he became an increasingly prominent figure within the FE group (highlighting the *social motive* for CT). Through the Facebook community, he became aware of the existence of an Elite that is trying to hide something from the general population. This constitutes the beginning of a fundamental distrust towards the media and institutions. P1 states “*I've kind of realized that this evil that's in the world, it's run by Freemasons and various other organizations*”. In agreement with Franks et al.’s (2017) findings, it should be noted here that the extreme skepticism and monological worldview is a late stage of conspiracy mentality rather than its defining feature from the outset.

According to P1 and many conspiracy theorists (see Franks et al., 2017), the outgroup is differentiated into three parts: First, there is a ‘powerful elite’ consisting of governments, wealthy families, and the media. To help the elite there is ‘middle management’, including university graduates, who theorize the world in such a way as to make the truth inaccessible to the rest of the population. The third group is the ‘ignorant population’ who simply don’t question anything. This outgroup classification not only functions to construct P1 as being part of an enlightened ingroup but also helps him to identify whom he can trust and what information is reliable. Thus, he cannot trust academics, media, and political systems, which is a classic feature of conspiratorial thinking (Aupers & Harambam, 2018). The following statement captures this distrust:

*“If you woke up to Flat Earth, if you woke up to someone being able to make up a lie as big as the Earth being round, that's just insane...That's the whole education system being infected in some way. The moon landing is a story in itself, but it is 100% false, I am 100% convinced of that. If governments can lie about the moon landing and satellites and the shape of the earth, well, they can lie about viruses and keep us in some kind of captivity for a whole year now”*

P1 is particularly critical of COVID-19 and believes that the Danish government is part of the Illuminati network, which aims to make the general public sick using vaccines. Furthermore, he points out that 9/11 was an inside job, a conclusion he has drawn based on evidence presented in YouTube videos. He further explains that while he doesn’t trust mainstream media, he considers FOX News and selected YouTubers to be reliable, and gets his information through these sources.

In addition, P1 places strong emphasis on conducting his own experiments; during the interview, he describes the numerous experiments he has done and repeatedly refers to himself as a ‘scientist’. Among other things, he uses infrared cameras, the Earth Curve Calculator and tracks ships via an app, all of which seem to speak in favor of a flat earth. P1 makes the case that very few people actually understand mathematics, and therefore it has been easy for the elite to trick people into believing that the earth is round. Based on some of these experiences P1 makes several appearances in newspapers and on TV, where he eagerly explains his experiments and promotes the FE theory.

Things started to go downhill for P1 as he experienced conflict in the Facebook group, which resulted in him no longer being allowed to be the administrator and ultimately the group broke up. Here he lost some of the recognition he otherwise gained, which constituted the social motive. At the same time as his belief in FE had been reinforced, he developed a strained relationship with his son, who could not reconcile himself with his father's beliefs. In the end, P1 has no contact with his son and has only seen his grandchild once. P1 expresses sorrow for this, but resolutely maintains that the FE theory is right for him. Thus, the social motive that FE filled in the beginning ends up being lost, as P1 neither has close relationships in the FE community nor with his family (but does distinguish himself as a scientist).

It can be said that P1 has sacrificed personal relationships for the sake of his deep investment in the FE theory. Ultimately, the theory seems to mainly serve an *existential motive* for him in making life meaningful. P1 states that the Big Bang theory is not true, as it would mean that mankind is an accidental creation of stardust: “*You are just a coincidence, you are stardust, rather than you being the center of the universe and having something to live for''*. The FE theory, on the other hand, puts man at the center, and provides a unified explanation of existence.

### Participant 2

P2 is a carpenter from the UK in his late 30’s. He lost his wife shortly after she gave birth to their third child (a *low point*). He begins the interview by describing himself as an average person, and elaborates that he has ceaselessly felt like an underdog as he could not compete academically with his peers. P2 has always been interested in CTs, and shared this fascination with his wife. The loss caused P2’s worldview to change in response to his feelings of unfairness, fear, and uncertainty (an *existential motive*). Shortly after she passed away P2 was scrolling on Facebook and saw a post by one of his old classmates about FE. He describes his surprise that some people believed in the theory, but the post sparked his interest. He began doing his own experiments and calculations. Wood and Douglas (2018) would describe these actions as an attempt at *compensatory control*, in which P2 searches for something static and controllable following the unforeseen tragedy.

After about a year of research, P2 was a firm believer of the FE theory, saying the newfound knowledge provided him with a better understanding of the world (an *epistemic motive*). His fear of the unpredictable is replaced by a new truth, marking a major *turning point* in his life. P2 explains how he noticed that there were discrepancies between his own calculations and the scientific explanation of gravity. As described by Moscovici (2008), this can be viewed as an effort to convert complex science to a more concrete, tangible, and relatable object. Further, he points out that he began to spot mistakes and lies in information provided by NASA.

“*The amount of NASA videos I’ve watched, where like, whether it is CGR or green screen or whatever it is that they are doing you can see the false in them, you can see the wires that they are hanging from, it almost looks comical now to watch it because I sit there and like point it all out*”.

P2 further describes that NASA acts as a part of middle management alongside celebrities and the royals, and that their purpose is to serve the elite. The elite consists of one family who controls the whole world. Additionally, P2 argues that we are all caught in a matrix that is impossible to escape and our destiny is to serve the elite. People who are unaware of this and don’t rebel against the elite, he classifies as ‘sheep’. As with P1, these categorizations distinguish between the different groups in society and help identify who can be trusted (Markova, 2016). When asked if he would ever reconsider his beliefs, P2 answers that he would never be able to unsee the truths that have presented themselves to him during the last years of research. He further elaborates on the hidden interconnection of events, a defining characteristic of conspiracy mentality:

*“Lots of big things are distractions, it’s like this thing with the boat in the Suez Canal. What do you think is going on with that? Do you think it is just a boat that accidentally crashed? Or do you think that was done on purpose to stop food coming through, or as another conspiracy says that it's full of trafficking children? And if you look up Hillary Clinton, was her cover name in all of the 3000 emails that got leaked? again more coincidences, it’s just, yeah, it’s a bit hard not to see it anymore.”.*

Unlike P1 who was an activist for FE, P2 dedication to the FE theory does not seem to affect his personal relationships much. He states that he has had a few heated discussions about FE with friends, but he never fell out with anyone: *“People did call me a bit of a crank or a nutjob whatever, but I don’t take that to heart”.* Additionally, he explains that his daughter is interested in his worldview, while his son is more concerned with his Xbox. He also makes an effort to emphasize that people are entitled to believe what they want, and that it is not his wish to push his belief down anyone’s throat. Rather he encourages us to look into the evidence ourselves and see which conclusions we draw from it.

P2 highlights that he does not consider himself religious, but is convinced there is a creator. Although he distances himself from religion, he mentions several religious elements throughout the interview. These elements consist of mythical stories, God, Satan, the soul and the Bible. P2 mentions that Disney, which is owned by the elite, places sexual objects in their movies with the purpose of “satanizing” the children watching them. Further P2 states that he does not know Satan by name, but that he is among us. The evidence for this is “the mark of the beast” which can be found on barcodes as well as in numerology. He takes note of the number 666, the number of the devil, as well as the number 33, which allegedly is a code for the elite, indicating that what is being said is a lie. P2 also narrates that there currently is an alien invasion taking place. These creatures are so-called “lizard people”, “reptiles” and “demons” and their purpose, in cooperation with the devil, is to take over our souls and raise the “antichrist”. The general mistrust towards the elite is especially clear in P2’s explanation: “*A lot of people ask the same question.’Why would they lie?’ and the short answer is that they want your soul. I can’t put it any blunter than that.*”

P2 continuously refers to the Bible and explains that he has come to the realization that some of its prophecies have come true. Prophecies are often seen in relation to CT, serving as a form of confirmation that tends to strengthen the belief (Robertson et al., 2018; Festinger et al. 1956). Despite P2's distance from religion, his narrative and explanations contain religious aspects. This is reflected in their references to the Bible and overt belief in God as a higher power and creator of the flat earth. The religious elements do not necessarily depend on the CT itself, but rather on the characteristics the individual believer attributes to the CT (Aupers & Harambam, 2018). Several key religious symbols are found in the narratives, linking abstract concepts to changes in the person’s understanding of the self (Obeyesekere, 1980; Riceour 1977).

P2 describes that though he feels trapped in the matrix, he is grateful for the knowledge FE has provided him: *“*In a way I am kind of thankful for it, because I’ve seen myself as a better person. I am not an angry person anymore. I take things in my stride. I’m not worried about what is going to happen. What will be will be, it is what it is*”*. Following Weber’s notion of ‘religious theodicy,’ we see how he has found calmness and meaning in his existence through CT.

### Participant 3

P3 is in his 50 s and works in the environmental field. He is from the US where he lives with his wife and daughter. Even before encountering FE theory, P3 speculated about the actual distance between the Sun, Moon, and Earth. While researching this topic, he came across a video explaining the distances based on FE theory. This caught his interest, and he began to explore it further by reading books and articles as well as watching YouTube videos. P3 explains that he chose to believe in the FE theory as the majority of the evidence presented was based on the Bible and therefore aligned with his beliefs. With a smile, P3 confides that he has not lived a sinless life but nevertheless has remained strong in his faith in God throughout his life. P3 opens up about a period in which he explored other religions but explains that he eventually returned to Christianity and has been a faithful believer ever since. P3's strong religious belief structures his general approach to life; FE is assimilated into this framework. Thus, CT is not uniquely used as a way to give meaning to life, but rather functions in combination with his already firm Christian belief.

It quickly becomes clear to us that P3 is skeptical of science, as he argues that gravity does not make logical sense and thus is not real.

*“And the gravity and all of that doesn't make sense either. Why hasn’t the moon been sucked into the earth after all these years? If gravity is so strong that it holds the atmosphere on the earth against the vacuum of space, but you can fly off of it and birds can fly, airplanes can fly, it just seems so bizarre the more you think about it to me”*

Unlike P1 and P2, P3 does not perform his own experiments. Instead, he trusts that other Flat Earthers conduct reliable research. He stands firm in his belief when we ask about the scientific explanation of gravity, explaining that he has no intention of changing his mind. This can be viewed as an example of confirmation bias (Casad, 2007), as P3 deliberately only takes arguments that support his views into account. Additionally, this could explain why P3 refers to Bible verses, which seem to confirm the FE theory. P3 continuously explains that society is divided into an outgroup with three distinct parts already discussed with P1 and P2. He sees the Elite’s main motive as hiding the existence of God to make it easier for them to control the rest of the population, who he categorizes as ‘sheep’. This is achieved by falsifying material from outer space, whereby the Earth appears round, and thus the Bible's descriptions of a flat Earth appear incongruous. P3 believes that middle management helps the Elite with their agenda and is responsible for the alleged faking of the moon landing by NASA. P3 gives the following example:

*“If you really look at the old spaceship from 1969 that thing doesn't look like it could go anywhere, you know. The little module that they say it was on the moon, and then back it's just unbelievable, if you reeeeally look at what they show that things made out of, it’s unbelievable that we fell for that”*.

P3 sounds discouraged as he explains that the sheep repeatedly believe the lies manufactured by the Elite and expresses a general distrust of public agencies and science. Additionally, he explains that he finds it important to make others aware of the Elite's agenda in order to prevent future lies from being accepted by the public:*“And in reality no, it doesn't really change the way we live if it's flat or round, it doesn't really change it. Then if you go to the next step who's running it and why are they lying to us and spending all that money “*. We ask whether he thinks the world situation will change if more people see through the elite and believe in God, to which P3 again refers to the Bible: He makes it clear that there is a predefined end to the world where everything will be destroyed and only those who believe in God will go to heaven. The problem, according to P3, is that a large part of the population does not believe in God, and thus will not experience the heavenly kingdom. The consistently clear division between the good and the bad is characteristic of religious stories (Oliver & Wood, 2014) and interacts with P3's FE belief. He concludes *'but in the end it's just a faith thing on that stuff. Will it make anything change? No."* Towards the end of the interview, we ask what impact the belief in FE has had on his life. P3 replies that the only significant change is that he is now confirmed in his earlier wonderings about the distance between the celestial objects; FE theory has provided the answers.

## Discussion

### A Distributed Model of Culture

Life-narrative studies such as the above lend themselves to a “distributed” model of culture, whereby collective cultural themes and symbols are selectively drawn upon and woven together in personal sense-making (Gregg, 1998). P1’s path to FE originated from a personal and economic crisis, where mathematical calculations, hands-on experiments and conspiratorial questions was something tangible and stable to hold on to. The same generally applies to P2, who experiences the loss of his wife overwhelming and intangible. Contrary to P1, P2 draws on both scientific and religious frameworks combined with conspiratorial questions about the society to develop his FE belief. A fundamental element for P1 and P2 concerns an existential motive that implies a need for control after an unforeseen misfortunate situation. The experience of a low point can thus be said to condition the following dedication to FE in the cases of P1 and P2. The situation is different for P3, who coincidentally encountered the theory through his existing religious belief and appears more assimilated in his belief in FE compared to P1 and P2. All participants explicitly stated that they considered the FE theory irrational at the beginning. However, it slowly sparked an interest, which later gave rise to a new understanding of the world, which helps each of them to fulfill both social, epistemic and existential motives. Through their belief in FE, P1, and P2 gain a coherent sense of meaning with life and the world they live in. For P3 the FE theory and religion work together, affirming and providing him with a cohesive explanation of the world. This explanation makes the world seem more tolerable and meaningful. P3 uses religious arguments in understanding FE, while P1 and P2 make more of their own calculations and rely on scientific arguments and rejects explanations and evidence that contradict FE theory of a flat Earth.

From the cases, it appears that all three participants each form their own worldview by drawing on other knowledge systems. This includes YouTube videos, scientific studies performed by themselves and others, religious elements, statements from the media, as well as questioning conspiratorial social structure and individual life circumstances. The life narrative analysis indicates that our participants select and refashion different elements and integrate them into their vision of reality. They become features of their personal culture (Gregg, 1998; Valsiner, 2014). The three life-narratives thereby show how the understanding and argumentation for the earth being flat has not arisen in an echo chamber, but instead draws on existing cultural and social elements: conspiratorial key symbols, personal models and calculations, religious elements, a general skepticism towards the structure of society, but also the social setting in which the people who believe in FE find themselves.

### A Paradoxical Minority: The Effects of Distrust

The self is constructed in interaction with other people. FE emerges as a community that shares the same ontological understandings which differs radically from the majority’s understanding of cosmology and social structure. This makes it interesting to discuss the extent to which Flat Earthers understand themselves as part of a community and how group affiliation influences their self-understanding. Flat Earth Society (FES) has received media exposure through the Netflix documentary "Behind the Curve". Based on this, it would be reasonable to assume that group affiliation contributes to the transition into a new cosmological and cultural understanding. However, preliminary empirical research showed the importance of distinguishing between FES and FE, as all our participants did not want to be associated with the FES. At the forefront of FES is YouTube icon Mark Sagent, who has created a big online following centered around the FE belief and him as a person. The community comes together online and at real-life conventions to discuss their beliefs. There are two reasons why our participants have expressed no interest in joining this community: first, as a member of a group you lose your autonomy; and second, their high skepticism contributes to a general mistrust of all people.

One of the challenges of joining a minority group is that you are held accountable for the actions and opinions of other members. This creates a paradox for the individual, as they wish to fulfill their basic need of belonging, but on the other hand, want to be seen as an individual with their own autonomy. With a prominent figure like Mark, other members must vouch for the statements and self-promotion made by him. P1 is very critical and describes FES as a "controlled opposition" as he believes they are spreading lies online about how the rest of the world should understand FE theory. He does not agree with their understanding of gravity, which supposedly emerges from the fact that the earth's disc moves upwards with one g-force. P3 describes how some FE'ers call Mark Sagent a "government plant" to make FE appear foolish. P2 states that he acknowledges the ability of FES to propagate arguments about a true witness, but that he does not find their image and the Netflix film credible. He strongly disagrees with their understanding of the earth as a disc. While the three participants in this study share a worldview they do not want to be identified as a group. Rather they appear as individuals who share common conceptions about the world. This finding nuances the description of an underlying social motive for conspiracy theorists (Douglas et al., 2017). P2 defines the FE as a 'community' that distances itself from 'society'.

The FE community has established a strong counter-pole to the majority in society. Due to its member’s high level of skepticism, FE is not a close-knit community. Breakwell (2021) proposes a distinction between distrust (the basis for removing or refusing trust has been established or decided), and mistrust (there is still uncertainty about whether trust is justified). On an intergroup level, FE’ers establish a general distrust by classifying society into three groups: the elite, middle management, and sheep – all of which play either conscious or unconscious roles in hiding the truth. Based on distrust, FE establishes a clear distinction between ingroup (FE) and outgroup (the majority). The ever-present suspicion towards other FEers remains, as one never fully decides for or against (Breakwell, 2021). P1, P2, and P3 all experience this state of active uncertainty at an ingroup level, as their FE ontology depends on individual models and calculations. P2 was called “stupid” when he asked clarifying questions in a FE Facebook group and was told to make his own calculations. P1 is also in favor of each individual believer doing their own calculations, as he felt no one could be trusted. The high level of skepticism creates a general level of mistrust towards society as a whole, as well as within the FE community.

### From a narrow to a broad approach to the study of CTs

In the previous discussion, it appears that none of the participants wants to be associated with a spokesperson in a particular group. The participants explain a desire to share nuances in their understanding of worldviews. More recent research argues that ‘CTs’ and ‘conspiracy theorists’ are not neutral terms, but rather function as categories to exclude from the field of knowledge (Dyrendal et al., 2018; Douglas et al., 2019). In accordance Bartich et al. (2018) argue that labels work as a way to shut out these people from the public debate. Both proclaim that the categorizations imply irrationality, paranoia, denial, and anti-science. The labels can thus delegitimize the theories and people believing in them (Dyrendal et al. 2018). We are aware of these connotations and that academics may contribute to these categorizations. However, in this study we find that the labels do not necessarily hold the connotations as suggested by the researchers. In an effort to counter stigmatization, we asked each of our participants if there were any particular terms they preferred us not to use. None of our subjects had any objections, and during the interviews, it became clear that they themself used ‘conspiracy theorists’, ‘conspiracy theory’, and ‘Flat Earther’ when referring to themselves and their beliefs. Thus, by engaging in open conversations and letting the subjects narrate and label their own identity and belief we were able to avoid some of the potential stigmatization. However, our findings support the study of Haramban and Aupers (2015), which suggests that it is essential for social science to be aware of the dialogical relationship between social science and CTs. If researchers are not careful in clarifying the difference between theoretical constructs and reality, research may then unintentionally contribute to a social stigmatizing of CTs. This can ultimately create an even greater gap between social science and conspiracy theorists (Deschrijver, 2021), and can potentially generate an fight for epistemic authority between science and CTs. This notion is important to have in mind when studying the phenomenon of CTs.

## Conclusion

This paper examined how a cultural psychological approach can unfold belief systems related to a person as a complex psychological system within a complex cultural system. Our empirical study has contributed to show how different systems of belief coalesce in FEers. FEers draw on both scientific arguments, religious elements as well as conspiratorial questions about the social structure. To expand the current understanding of individuals behind FE beliefs and to provide a nuanced theoretical perspective on CT thinking, we have employed an integrative methodology. This approach suggests that individual, social, historical, and sociocultural elements all play a role in shaping the worldviews of FEers and conspiracy theorists in general. In accordance with other studies, our study suggests that peoples’ need to create a coherent and cohesive explanation of the world increases when the world is perceived as uncertain (Miller et al., 2016); CTs can offer epistemic explanations as well as fulfill existential and social desires (Douglas et al. 2017); CT beliefs are to a greater extent a response to a lack of epistemic trust in representative democracy and a desire to give more power to the people (Pantazi et al., 2022; Pierre, 2020). Moreover, we illustrate that this sense of meaning is attained through diverse pathways, influenced by each individual's unique life history and personal challenges. Thus a cultural psychological approach offers a comprehensive framework for understanding the complex and context-dependent nature of FE beliefs, as it allows us to explore how personal and cultural factors interact to shape individual worldviews, providing a deeper and more nuanced understanding than is typical in the study of CTs.

## Data Availability

The anonymised interviews can be shared upon request from readers but are not openly available in an online archive. Extensive case description and quotations are also provided in the manuscript.
